# Controlled attenuation parameter-insulin resistance (CIR) score to predict non-alcoholic steatohepatitis

**DOI:** 10.1038/s41598-022-25931-7

**Published:** 2022-12-19

**Authors:** Juan Macias, Pablo Parra-Membrives, Francisco Sosa-Moreno, Pilar Rincon, Dario Martinez-Baena, Marta Fernandez-Fuertes, Jose M. Lorente-Herce, Rafael C. Martinez, Granada Jimenez-Riera, Anaïs Corma-Gomez, Alejandro Gonzalez-Serna, Juan A. Pineda, Luis Miguel Real

**Affiliations:** 1 Departamento de Medicina, Universidad de Sevilla, Sevilla, Spain; 2 Unit of Infectious Diseases and Microbiology, Hospital Universitario Virgen de Valme, Avda Bellavista Sn, 41014 Sevilla, Spain; 3 CIBER de Enfermedades Infecciosas (CIBERINFEC), Instituto de Salud Carlos III, Madrid, Spain; 4 Instituto de Biomedicina de Sevilla (IBiS), Sevilla, Spain; 5 Unit of Hepatobiliary and Pancreatic Surgery. Service of General and Digestive Surgery, Hospital Universitario Virgen de Valme, Sevilla, Spain; 6 Unit of Pathology, Hospital Universitario Virgen de Valme, Sevilla, Spain; 7 Departamento de Fisiología, Facultad de Farmacia, Universidad de Sevilla, Sevilla, Spain; 8 Departamento de Especialidades Quirúrgicas, Bioquímica e Inmunología, Universidad de Málaga, Málaga, Spain

**Keywords:** Non-alcoholic fatty liver disease, Non-alcoholic steatohepatitis

## Abstract

The diagnosis of non-alcoholic steatohepatitis (NASH) requires liver biopsy. Patients with NASH are at risk of progression to advanced fibrosis and hepatocellular carcinoma. A reliable non-invasive tool for the detection of NASH is needed. We aimed at developing a tool to diagnose NASH based on a predictive model including routine clinical and transient hepatic elastography (TE) data. All subjects undergoing elective cholecystectomy in our center were invited to participate, if alcohol intake was < 30 g/d for men and < 15 g/d for women. TE with controlled attenuation parameter (CAP) was obtained before surgery. A liver biopsy was taken during surgery. Multivariate logistic regression models to predict NASH were constructed with the first 100 patients, the elaboration group, and the results were validated in the next pre-planned 50 patients. Overall, 155 patients underwent liver biopsy. In the elaboration group, independent predictors of NASH were CAP value [adjusted OR (AOR) 1.024, 95% confidence interval (95% CI) 1.002–1.046, p = 0.030] and HOMA value (AOR 1.847, 95% CI 1.203–2.835, p < 0.001). An index derived from the logistic regression equation to identify NASH was designated as the CAP-insulin resistance (CIR) score. The area under the receiver operating characteristic curve (95%CI) of the CIR score was 0.93 (0.87–0.99). Positive (PPV) and negative predictive values (NPV) of the CIR score were 82% and 91%, respectively. In the validation set, PPV was 83% and NPV was 88%. In conclusion, the CIR score, a simple index based on CAP and HOMA, can reliably identify patients with and without NASH.

## Introduction

Non-alcoholic fatty liver disease (NAFLD) is the most common liver disorder in western industrialized countries, where the main risk factors for NAFLD are more common, such as central obesity, type 2 diabetes mellitus (T2DM), dyslipidemia, and the metabolic syndrome^1^. Worldwide, the prevalence of NAFLD is around 20%, with higher frequencies in western countries^1^. In particular subpopulations, however, it reaches even more alarming proportions. Thus, in patients with morbid obesity, T2DM or infected by the human immunodeficiency virus, the frequency of NAFLD amounts to approximately 40%^1–3^. Because of the potential of NAFLD to progress to non-alcoholic steatohepatitis (NASH), which increases the risk of cirrhosis and hepatocarcinoma, it is considered that NAFLD will emerge as the leading cause of liver disease in western countries in the near future^1^.

The diagnosis of NASH, to date, involves a histological evaluation of the liver through biopsy^4^. However, liver biopsy has multiple limitations. It is invasive and not free of risks and serious complications. The inter-observer and intra-observer variabilities can be severe and highly dependent on the size of the liver sample obtained by biopsy^4^. Furthermore, a screening technique for a prevalent disease in the general population cannot be invasive, as it is the case with liver biopsy. Finally, changes in the degree of simple steatosis and its progression to NASH cannot be conveniently monitored using repeated biopsies over time. A reliable non-invasive tool for screening and following the progression to NASH over time in very large population samples is necessary.

Non-invasive tests have been developed to predict the presence of simple steatosis. Blood test panels developed to diagnose NASH have poor positive (PPV) and negative (NPV) predictive values^5,6^, which prevent their translation into clinical use. Imaging techniques are the most specific tests to diagnose steatosis. Proton nuclear magnetic resonance imaging (MRI) spectroscopy or fat signal subtraction MRI have greater sensitivity and specificity, but their interpretation is complicated and requires a long period of immobility in a closed space^7,8^. The controlled attenuation parameter (CAP) is a measurement obtained with commercial hepatic transient elastography devices^9^. This is a point-of-care technique which has been developed in different groups of patients and has been validated independently^9–12^. It is able to reliably predict the presence of simple steatosis^9–12^. Furthermore, the technique is reproducible, with small inter-observer variability^13^. Unfortunately, none of the techniques mentioned can differentiate simple steatosis from NASH and this makes liver biopsy is still necessary^4^. Due to these, our aim was to develop a tool to diagnose the presence of NASH based on a predictive model including routine clinical data and information based on transient hepatic elastometry.

## Patients and methods

### Design and patients

This was a prospective cross-sectional study carried out in a single center from April 2015 to December 2018. All subjects undergoing elective cholecystectomy were offered to participate in the study if they met the following inclusion criteria: (1) Willingness and ability to give their written informed consent; (2) Age ≥ 18 years; (3) Male or female not pregnant or lactating; (4) Caucasian white race; (5) Average daily alcohol consumption < 30 g in men and < 15 g in women. The following subjects were excluded from the study: (1) Liver disease, including tumor, vascular, autoimmune, infectious or metabolic liver diseases other than NAFLD; (2) Preoperative risk according to the American Association of Anesthetists ≥ 4; (3) Coagulation disorders; (4) Complications throughout the cholecystectomy that made it not advisable to continue with the liver biopsy protocol; (5) History of a malignant neoplasm.

### Liver biopsy

Liver samples were obtained by means of at least one deep biopsy in the course of elective cholecystectomy. A single pathologist (R.C.M), blinded to clinical data, reviewed all biopsies. For each biopsy, a preestablished form for evaluation of the main histologic patterns was filled out by the pathologist. The histological check‐list was from the Steatosis, Activity, Fibrosis (SAF) score^14^.

For each patient, the SAF score was calculated as follows^14^: (1) The steatosis score (S) assessed the quantities of large or medium‐sized lipid droplets, but not foamy microvesicules, from 0 to 3 (S0: < 5%; S1: 5%‐33%, mild; S2: 34%‐66%, moderate; S3: > 67%, marked); (2) Activity grade (A, from 0‐4) was the unweighted addition of hepatocyte ballooning (0‐2) and lobular inflammation (0‐2). Ballooning was graded from 0 to 2 as follows: grade 0: normal hepatocytes; grade 1: presence of clusters of hepatocytes with a rounded shape and pale cytoplasm usually reticulated, with size similar to that of normal hepatocytes; grade 2: same as grade 1 with some enlarged hepatocytes, at least twofold that of normal cells. Lobular inflammation was defined as a focus of two or more inflammatory cells within the lobule. Foci were counted at 20×magnification (0: none; 1: ≤ 2 foci per 20× ; 2: > 2 foci per 20×); (3) Stage of fibrosis (F) was assessed using the following score: Stage 0 (F0): none; stage 1 (F1): perisinusoidal zone 3 or portal fibrosis, stage 2 (F2): perisinusoidal and periportal fibrosis without bridging, stage 3 (F3): bridging fibrosis and stage 4 (F4): cirrhosis.

NAFLD was defined by the presence of steatosis in > 5% of hepatocytes, and NASH by the presence, in addition, of hepatocellular ballooning of any degree and lobular inflammatory infiltrates of any amount.

### Clinical data collection

Within the four weeks prior to obtaining the liver sample, the following variables were collected: Age, sex, body mass index (BMI), diagnosis of T2DM and blood pressure. A fasting blood sample was drawn within one week before the liver biopsy for the following determinations: Triglycerides, total cholesterol, HDL-cholesterol, LDL-cholesterol, glycemia, ALT, AST, GGT. In addition, part of the sera was stored and frozen at − 80ºC to determine fasting insulin. The insulin resistance was determined by means of the homeostasis model assessment (HOMA). HOMA was calculated according to the following equation: fasting insulin (µU/mL) × fasting glucose (mmol/L)/22.5. The metabolic syndrome was defined according to the NCEP ATP III if three or more of the following criteria were met: (1) Waist circumference > 102 cm (men) or > 88 cm (women), (2) Blood pressure ≥ 130/85 mmHg or drug treatment for hypertension, (3) Fasting triglyceride level ≥ 150 mg/dL or treatment for high triglyceride level, (4) Fasting HDL-cholesterol level < 40 mg/dl (men) or < 50 mg/dl (women) or treatment for low HDL-cholesterol level, and (5) Fasting blood sugar ≥ 100 mg/dl.

### Liver stiffness and controlled attenuation parameter

Determinations of CAP and liver stiffness were obtained by means of a commercial transient elastography device with the standard M probe (FibroScan 502, Echosens, Paris, France). An experienced researcher made all elastographic determinations within four weeks of liver biopsy sampling. Patients were required to be fasting. The measurements were considered valid when at least 10 valid acquisitions were obtained, with an interquartile range (IQR) < 40 dB/m and a success rate > 60%^15^. The CAP value of < 238 dB/m was applied to rule out steatosis involving ≥ 10% of hepatocytes^11,12^.

### Statistical analysis

The main outcome variable was the presence of NASH, histologically diagnosed according to the SAF criteria. A univariate analysis was performed to determine the associations between the main outcome variable, the presence of NASH in liver tissue, and the following independent variables: Age, sex, BMI, T2DM, triglycerides, total cholesterol, HDL-cholesterol, LDL-cholesterol, fasting blood glucose, HOMA, ALT, AST, GGT, liver stiffness and CAP value. Comparisons between categorical variables were done applying the Chi-square test or the Fisher test. Comparisons between continuous variables were carried out using the Student *t* test or the Mann–Whitney-*U* test. For comparisons among more than two independent groups, the Chi-square test was used for categorical variables and the Kruskal–Wallis test for continuous variables. Those independent variables with a level of association with NASH with a value p ≤ 0.1 were included in logistic regression models, adjusted by age and sex. The best fitted model was selected to use the corresponding regression equation to predict the presence of NASH. The diagnostic performance of the model was examined by means of receiver-operating characteristic (ROC) curves. The area under the ROC curve (AUROC) and the 95% confidence interval (95% CI) of the AUROC were calculated. The point with optimal positive predictive value (PPV) and negative predictive value (NPV) was chosen from the ROC curve to diagnose and rule out, respectively, the presence of NASH. For the development of a predictive model of NASH, the first 100 consecutive study participants were selected. The performance of the model was planned to be validated in the next 50 consecutive patients. In addition, two cut-offs points were selected from the ROC curve to maximize PPV and NPV. For the statistical analysis, the statistical package IBM SPSS 25 (SPSS Inc. IBM) was used.

### Ethical issues

The study complied with the Helsinki declaration and was approved by the Hospital Virgen de Valme Ethics Committee (0069-N-15). All patients gave their written informed consent to be included in the study.

### Patient and public involvement

Patients or the public were not involved in the design, conduct, reporting or dissemination plans of this study.

### Ethics approval

The study was conducted according to the guidelines of the Declaration of Helsinki, and approved by the Hospital Virgen de Valme Ethics Committee (0069-N-15). Informed consent was obtained from all subjects involved in the study.

## Results

### Characteristics of the study population

**Figure 1:**
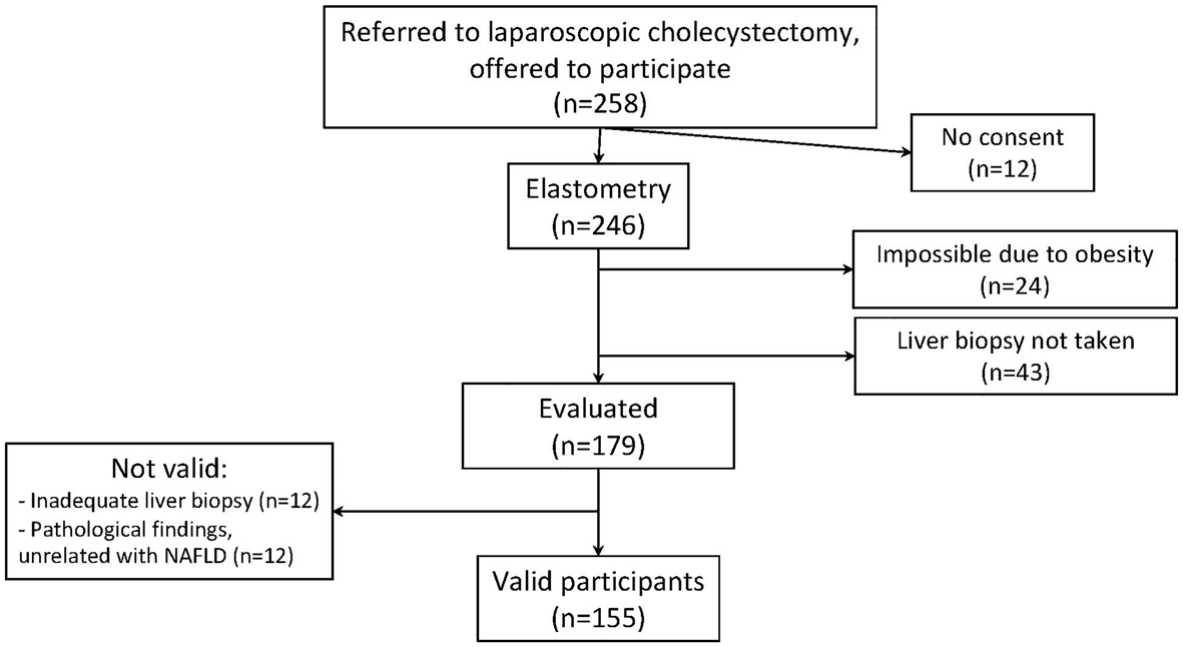
Disposition of patients.

Overall, 258 patients were offered to participate in the study, 12 (4.7%) of them did not consent to participate. Thus, 246 individuals were included in the study. Elastography yielded unreliable measurements or no measurement was obtained in 24 (9.8%) patients due to obesity. Among 222 subjects with full study pre-surgical evaluation, 43 (18%) patients did not undergo a liver biopsy due to decisions of surgeon during cholecystectomy. The characteristics of those patients are summarized in Table 1. Of 179 patients with liver biopsy, 13 (7.3%) had liver samples deemed as inadequate for the study, and 13 (7.3%) had unexpected pathological findings, unrelated with NAFLD. Finally, 155 patients were evaluable for the study. There were no significant differences between these 155 evaluable patients and those 43 without liver biopsy (Table 1). The disposition of patients is summarized in Fig. 1.

**Table 1:** Characteristics of the patients included and excluded due to lack of liver biopsy.

Characteristics	Valid patients (n = 155)	Patients without liver biopsy (n = 43)	p
Age, years†	61 (49–71)	65 (51–73)	0.488
Female sex, n (%)	97 (67)	23 (54)	0.280
BMI, Kg/m^2†^	28 (26–32)	28 (24–31)	0.350
T2DM, n (%)	30 (19)	6 (14)	0.417
Glycemia, mg/dL^†^	96 (87–108)	95 (89–107)	0.809
HOMA^†^	2.7 (1.5–3.98)	2.6 (1.8–3.9)	0.586
Triglycerides, mg/dL^†^	108 (80–148)	108 (76–176)	0.656
Cholesterol, mg/dL^†^	192 (172–219)	187 (164–226)	0.419
HDL-cholesterol, mg/dL^†^	51 (42–59)	53 (44–63)	0.547
Metabolic syndrome, n (%)	103 (67)	28 (65)	0.870
ALT, UI/mL^†^	18 (14–27)	19 (15–26)	0.545
AST, UI/mL^†^	18 (16–23)	18 (15–24)	0.246
GGT, UI/mL^†^	32 (22–62)	31 (18–56)	0.510
Liver stiffness, kPa^†^	5.1 (4.1–6.6)	4.8 (4.2–5.3)	0.279
CAP, dB/m^†^	264 (233–308)	266 (219–327)	0.485

*BMI* body mass index, *T2DM* type 2 diabetes mellitus, *HOMA* homeostasis model assessment.

^†^Median (Q1-Q3).

### Liver biopsy

Among the evaluable patients, the median (Q1–Q3) length of the liver biopsy samples was 21 (15–32) mm. Following the SAF algorithm, 83 (54%) patients were classified as S0 (Supplementary Fig.). In these patients without steatosis, one (1.2%) had ballooning grade 1, and 22 (27%) had lobular inflammation. Fibrosis stage F1 was identified in 16 (19%) patients without steatosis. Of the group without NAFLD, 51 (62%) patients did not show ballooning, lobular inflammation nor fibrosis, thus presenting normal liver histology.

Among 72 (47%) patients with NAFLD, 28 (38%) patients were classified as NASH (Supplementary Fig.). Fibrosis was observed in 22 (79%) patients with NASH: F1, 14 (50%); F2, 4 (14%), F3, 3 (11%); F4, 1 (3.6%). The 44 patients with NAFLD without NASH included 21 (48%) patients only with inflammation or ballooning, and 30 (68%) without fibrosis. Fibrosis F1 and F2 were identified in 11 (25%) and 3 (6.8%) patients with NAFLD without NASH, respectively.

### Associations with SAF category

Variables associated with SAF category are summarized in Table 2. Metabolic factors as BMI, T2DM, fasting glycemia, plasma triglycerides, HDL-cholesterol and HOMA were significantly associated with the SAF category. CAP values were higher for patients with NAFLD without NASH compared to those without steatosis (p < 0.001). Individuals with NASH had CAP values significantly greater than patients with NAFLD without NASH (p < 0.001).

**Table 2:** Variables associated with the SAF category: No NAFLD, NAFLD without NASH and NASH (n = 155).

Variable	No NAFLD (n = 83)	NAFLD no NASH (n = 44)	NASH (n = 28)	p
Age, years	59 (44–70)	61 (54–71)	66 (54–71)	0.099
Female sex, n (%)	52 (63)	26 (59)	19 (68)	0.755
BMI, Kg/m^2†^	26.9 (24–29)	28.9 (25–33)	30.7 (27–35)	0.004
T2DM, n (%)	9 (11)	4 (9)	17 (61)	< 0.001
FPG ≥ 100 mg/dL, n (%)	22 (27)	23 (52)	22 (79)	< 0.001
FPG, mg/dL^†^	91 (85–101)	101 (92–107)	112 (101–135)	< 0.001
HOMA^†^	1.8 (1.1–2.98)	2.9 (1.9–3.3)	5.6 (3.9–9.0)	< 0.001
Triglycerides, mg/dL^†^	98 (67–118)	110 (83–153)	132 (95–218)	0.001
Cholesterol, mg/dL^†^	189 (164–214)	194 (187–236)	191 (173–233)	0.138
HDL-c, mg/dL^†^	53 (43–65)	48 (40–64)	45 (35–57)	0.009
Metabolic syndrome, n (%)	50 (52)	36 (78)	23 (82)	0.001
ALT, IU/mL^†^	16 (13–25)	21 (15–27)	22 (17–34)	0.018
AST, IU/mL^†^	27 (15–23)	19 (16–22)	20 (16–26)	0.220
GGT, IU/mL^†^	27 (17–65)	34 (26–88)	48 (31–82)	0.001
Liver stiffness, kPa^†^	4.6 (3.8–6.3)	5.6 (4.3–6.5)	7.0 (4.9–11.7)	< 0.001
CAP, dB/m^†^	239 (204–268)	287 (263–322)	337 (279–365)	< 0.001

*CAP* controlled attenuation parameter, *BMI* Body mass index, *HDL-c* HDL-cholesterol, *HOMA* homeostasis model assessment, *FPG* Fasting plasma glucose, *T2DM* type 2 diabetes mellitus.

^†^Median (Q1-Q3).

### Model to predict NASH

The characteristics of both the elaboration and validation groups are summarized in Table 3. There were no significant differences between the elaboration and the validation groups in variables potentially associated with NASH (Table 3). In the construction of the model, the only variables independently associated with NASH were HOMA and CAP value (Table 4). The equation of a model restricted to those variables was 1/(1 + e^Y^), where Y = 9.17 + 0.018*CAP + 0.515*HOMA. This index derived from the logistic regresion equation to identify NASH was designated as the CAP-insulin resistance (CIR) score. The AUROC (95% CI) of the CIR score was 0.93 (0.87–0.99). The cut-off point of 0.569 was selected for optimal predictive values. Applying this cut-off, 9 of 11 patients with NASH were correctly classified, and 81 of 89 patients without NASH were correctly identified. Thus, the CIR score yielded a PPV of 82% and a NPV of 91%. This cut-off was validated in the remaining 55 patients. In the validation set, the PPV was 83% (n/N = 5/6 patients with NASH correctly classified) and the NPV was 88% (n/N = 43/49 patients without NASH correctly classified).

**Table 3:** Characteristics of the elaboration set, first 100 patients, and the validation set, remaining 55 patients.

Characteristics	Elaboration group (n = 100)	Validation group (n = 55)	p
Age, years^†^	62 (51–71)	60 (46–67)	0.186
Female sex, n (%)	61 (61)	36 (66)	0.583
BMI, Kg/m^2†^	28 (26–32)	28 (25–31)	0.893
T2DM, n (%)	18 (18)	12 (22)	0.565
Glycemia, mg/dL^†^	95 (87–107)	96 (89–108)	0.731
HOMA^†^	2.4 (1.5–4.1)	3.0 (1.6–3.7)	0.607
Triglycerides, mg/dL^†^	105 (80–150)	116 (79–141)	0.722
Cholesterol, mg/dL^†^	193 (172–219)	191 (174–214)	0.659
HDL-cholesterol, mg/dL^†^	51 (42–61)	51 (42–58)	0.547
Metabolic syndrome, n (%)	68 (68)	35 (64)	0.582
ALT, UI/mL^†^	18 (14–27)	17 (15–24)	0.659
AST, UI/mL^†^	19 (16–23)	18 (15–24)	0.337
GGT, UI/mL^†^	33 (22–65)	31 (18–53)	0.283
Liver stiffness, kPa^†^	5.2 (4.2–6.4)	4.8 (3.8–7.2)	0.355
CAP, dB/m†	267 (233–322)	258 (232–301)	0.353
**SAF score, n (%)**			
No NAFLD	52 (52)	31 (56)	0.612
NAFLD without NASH	31 (31)	13 (24)	
NASH	17 (17)	11 (20)	

*BMI* Body mass index, *T2DM* type 2 diabetes mellitus, *HOMA* homeostasis model assessment.

^†^Median (Q1-Q3).

**Table 4:** Model to predict NASH (elaboration set, n = 100).

Variable	N	NASH N (%)	P univariate	Adjusted Odds Ratio (95% confidence interval)	Multivariate p
**Age**^†^			0.183*	1.033 (0.983–1.098)	0.202
< 62 years	50	6 (12)			
≥ 62 years	50	11 (22)			
**Sex**			0.374		0.346
Female	61	12 (20)		1.845 (0.501–7.170)	
Male	39	5 (13)			
**BMI**^‡^			0.055*	1.002 (0.852–1.179)	0.978
< 30 kg/m^2^	67	8 (12)			
≥ 30 kg/m^2^	33	9 (27)			
**HOMA**^§^			< 0.001*	2.221 (1.501–2.286)	< 0.001
< 4	25	3 (4)			
≥ 4	75	14 (56)			
**Triglycerides**^§^			0.026*	0.992 (0.979–1.005)	0.206
< 150 mg/dL	76	9 (12)			
≥ 150 mg/dL	24	8 (33)			
**HDL-c**^§^			0.002*	0.997 (0.943–1.055)	0.924
< 45 mg/dL	33	11 (33)			
≥ 45 mg/dL	67	6 (9)			
**Metabolic syndrome**			0.068	5.885 (0.788–43.962)	0.084
No	32	2 (6)			
Yes	68	14 (21)			
**AST**^§^			0.432	–	–
< 37 mg/dL	97	16 (17)			
≥ 37 mg/dL	3	1 (33)			
**GGT**^§^			0.115*	–	–
< 66 IU/mL	76	10 (13)			
≥ 66 IU/mL	24	7 (29)			
**Liver stiffness**^†^			0.062*	1.087 (0.826–1.432)	0.550
< 5.2 kPa	50	5 (10)			
≥ 5.2 kPa	50	12 (24)			
**CAP**^¶^			0.005*	1.016 (1.002–1.031)	0.021
< 248 dB/m	36	1 (2.8)			
≥ 248 dB/m	64	16 (25)			

*CAP* controlled attenuation parameter,* BMI* Body mass index,* HOMA* homeostasis model assessment.

*Entered in the logistic regression model as continuous variables.

Categories of continuous variables: ^†^By the median; ^‡^BMI ≥ 30 kg/ m^2^, indicative of obesity; ^§^Categorized by upper limit of normal; ^¶^:CAP ≥ 248 dB/m, cut-off for steatosis (18).

Using two cut-off points, ≥ 0.654 and ≤ 0.062, to maximize predictive values, 93 (60%) of the overall 155 study patients were classified using the CIR score. Among them, 78 of 78 individuals without NASH and 13 of 15 patients with NASH were correctly identified, yielding a PPV of 87% and a NPV of 100%.

## Discussion

The CIR score, a simple index combining a measure of insulin resistance, as HOMA value, and CAP measurements achieves a high diagnostic yield to predict NASH. Using two cut-off points, this model reaches a high accuracy to identify NASH and could rule out with certainty NASH.

The strongest predictor of NASH in the present study was HOMA. This finding was not surprising since insulin resistance in liver, muscle and adipose tissue is key in the development of NAFLD^16^. Overnutrition causes excess circulating fatty acids. Fatty acids and oxidated fatty acids accumulate in peripheral tissues, including liver and adipose tissue, resulting in insulin resistance. Some types of lipids that accumulate in NAFLD, as fatty acids, diacylglycerol or oxysterols, can injure hepatocytes^16^. In addition to its directly cytotoxic effects, fatty acid accumulation exacerbates insulin resistance and hyperinsulinemia, which leads to further hepatic lipid accumulation, and promotes inflammatory and fibrogenic responses^16^. Indeed, HOMA has been linked with histological features of NASH, as steatosis and ballooning, and progression of fibrosis in patients with NAFLD^17^.

The presence of steatosis is a necessary first step to diagnose NASH^4,14^. CAP is a technique for the measurement of steatosis within hepatic transient elastography. As expected, CAP value was associated with NAFLD in the present study and was a predictor of NASH. A meta-analysis of nine studies showed good sensitivity and specificity, and high diagnostic accuracy of CAP to detect steatosis^18^. One of the main issues of studies validating the use of CAP to predict steatosis was the heterogeneity of populations, with different etiologies of chronic liver diseases pooled along with NAFLD^22^. In fact, CAP interpretation was influenced by the etiology of liver disease in a meta-analysis on individual patient data^19^. To solve this issue, a recent study reported the diagnostic yield of CAP in patients who underwent liver biopsy for suspected NAFLD^20^. In the present study, individuals with diagnoses other than NAFLD were excluded, and only participants without liver disease or with NAFLD were included. In this setting of a homogeneous population, including individuals without NAFLD, CAP values showed a high correlation with the grade of steatosis and the SAF classification.

Herein, we found that the CIR score, a combination of CAP measurement and HOMA value, had a good diagnostic yield for NASH. Studies on the ability of elastography to discriminate between isolated steatosis and NASH are limited^21–23^. These studies focused on the non-invasive estimation of steatosis and fibrosis among patients with NAFLD. In one study, the addition of cytokeratin 18 to CAP and liver stiffness values in one study did not significantly improve the prediction of NASH^21^. In another study, a score to identify patients with a composed outcome of NASH, elevated NAFLD activity score, and fibrosis F2 or higher was developed^24^. Among patients referred to liver biopsy for suspected NAFLD, a combination of liver stiffness, CAP measurement and AST levels yielded a PPV of 83% and NPV of 85%^24^. These predictive values were not validated in any external validation groups^24^. Magnetic resonance imaging-based studies did not yield better results to identify NASH^21–23^. Currently, it is considered that neither hepatic transient elastography nor magnetic resonance imaging can reliably discriminate NASH from simple steatosis^4,25^. Herein, we report a simple index, the CIR score, that allows to exclude the presence of NASH with certainty, and to diagnose NASH with an acceptable rate of misclassifications. In addition, the CIR score was subject to a pre-planned internal validation. However, the application of this tool for individual patients needs external independent validation.

The application of the CIR score to select patients likely to suffer NASH, along with liver stiffness measurement, could facilitate the identification of candidates to drug therapy against NASH. Agents under development for NASH will target individuals with the most aggressive NASH variant, i.e. patients with NASH and fibrosis stage F2 or greater^26,27^. Clinical trials have focused on this group of patients because those with progression to significant liver fibrosis are at risk of liver events and liver related death^28^. The main issue will be the detection of these patients with NASH and fibrosis among the overall population with NAFLD, which in Western countries amounts to more than 20% of the general population^1^. The use of a simple non-invasive index, as the CIR score, could aid the screening large populations to identify the group at risk of NASH-related fibrosis progression. The inherent liver stiffness measurement during transient elastography could accurately identify those patients also harboring liver fibrosis ≥ F2.

The present study has some limitations. First, the use of liver biopsy as gold standard to diagnose NAFLD is far from perfect. This may have affected the classification of patients and, as a consequence, the diagnostic yield of the CIR score to predict NASH. Second, 18% subjects with full study pre-surgical evaluation did not undergo a liver biopsy during cholecystectomy. This relatively high proportion of candidates not included in the study could represent a selection bias. However, the characteristics of those patients did not significantly differ from those of the individuals included in the study. In addition, an XL probe was not available for our study. Elastography with an M probe yielded not valid measurements in nearly 10% patients. This failure rate is within the reported 4–24% failure rate for the M probe, but it could have been lower applying an XL probe^25^. Third, patients with gallstones are not representative of the general population. Gallstone disease and NAFLD may be linked by common risk factors, as obesity. The prevalence of NASH in patients referred to cholecystectomy in the present study is within the frequency of NASH in patients undergoing cholecystectomy in previous reports, ranging between 10 and 55%^29,30^. The diagnostic yield of the CIR score could change if applied to other populations with higher prevalence of NASH. On the contrary, the expected lower frequency of NASH in the general population could increase the NPV of the CIR score. In addition, this was a prospective elaboration of a non-invasive tool to predict NASH, with an internal validation. Clinical data, blood and imaging tests to construct the regression models were collected close to the liver sampling. Liver biopsies were taken during cholecystectomy, allowing for large histological samples, and were evaluated by a single experienced pathologist. These are strengths of the study.

In conclusion, the CIR score, a diagnostic tool that brings together a routine blood test and point-of-care imaging, allows the classification of patients as carriers or not of NASH. The CIR score could respond to the healthcare need to diagnose aggressive forms of NAFLD in a non-invasive and simple way. Moreover, along with simultaneous fibrosis assessment through liver stiffness measurement, the CIR score could allow the massive screening and detection of those patients in need of implementing treatment against NASH.

## Supplementary Information

Supplementary Figure 1.

## Data Availability

The data that support the findings of this study are available from the corresponding author upon request.
