# Supplementary figures and images for: Controlled attenuation parameter-insulin resistance (CIR) score to predict non-alcoholic steatohepatitis
Source: Sci Rep. 2022 Dec 19;12:21897. doi: 10.1038/s41598-022-25931-7 (PMC9763491; doi:10.1038/s41598-022-25931-7)

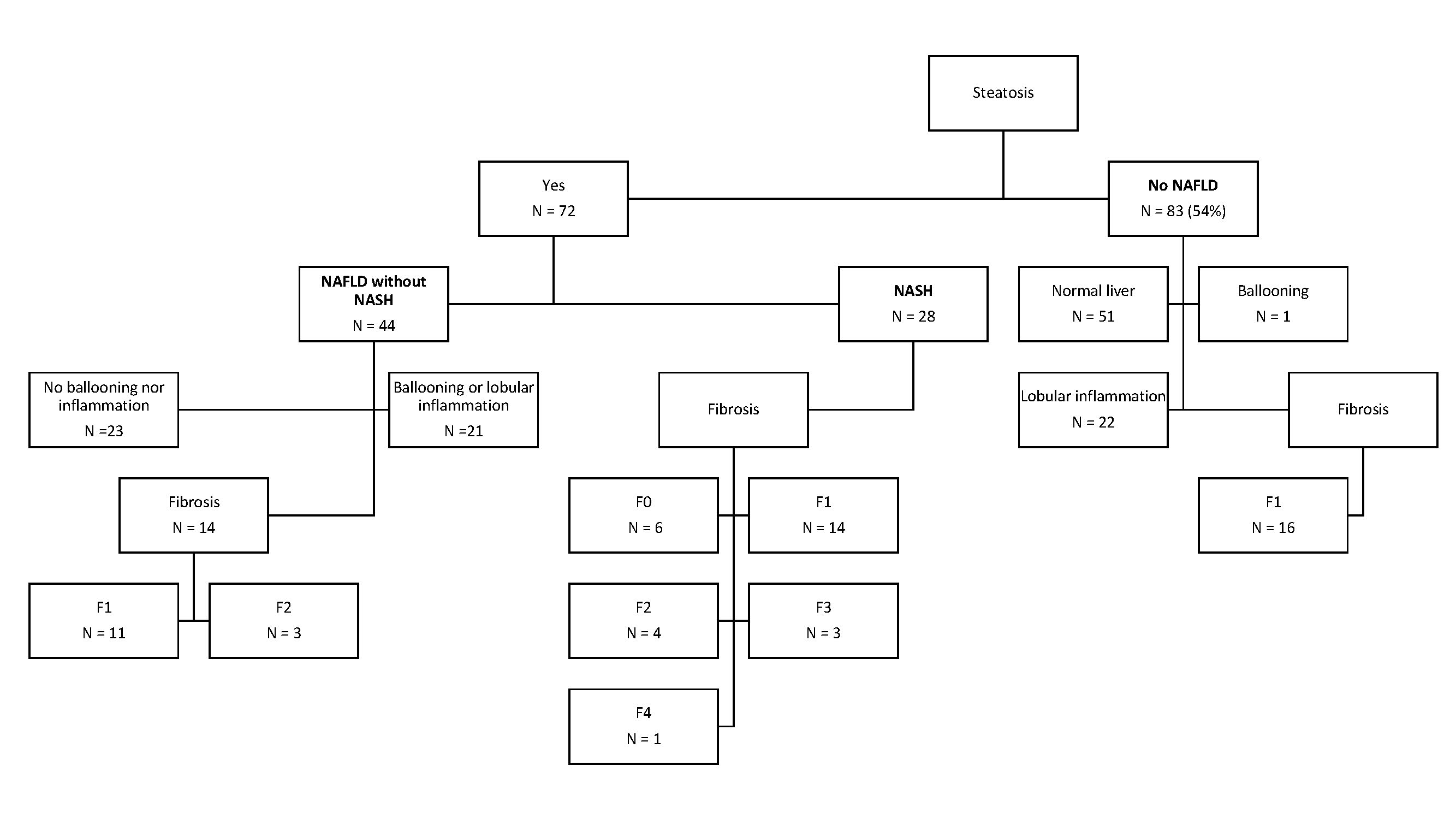

Supplement: Supplementary file 1 — Supplementary Figure 1. [file 41598_2022_25931_MOESM1_ESM.jpg]
